# Evaluation of Two Web-Based Interventions (Res-Up! and REMOTION) in Routine Outpatient Psychotherapy (Therapy Online Plus—TOP): Protocol for a Randomized Controlled Trial

**DOI:** 10.2196/41413

**Published:** 2023-03-15

**Authors:** Leonie Franziska Trimpop, Laura Luisa Bielinski, Thomas Berger, Ulrike Willutzki

**Affiliations:** 1 Department of Clinical Psychology and Psychotherapy University of Witten/Herdecke Witten Germany; 2 Department of Clinical Psychology and Psychotherapy University of Bern Bern Switzerland

**Keywords:** online therapy, randomized controlled trial, transdiagnostic, resilience, emotion regulation, capitalization, compensation, intervention, psychotherapy, Germany, treatment, mental disorder, effectiveness

## Abstract

**Background:**

Only 11%-40% of those with a mental disorder in Germany receive treatment. In many cases, face-to-face psychotherapy is not available because of limited resources, such as an insufficient number of therapists in the area. New approaches to improve the German health care system are needed to counter chronification. Web-based interventions have been shown to be effective as stand-alone and add-on treatments to routine practice. Interventions designed for a wide range of mental disorders such as transdiagnostic interventions are needed to make treatment for mental disorders more accessible and thus shorten waiting times and mitigate the chronification of mental health problems. In general, interventions can be differentiated as having either a capitalization (CAP) focus—thus drawing on already existing strengths—or a compensation (COMP) focus—trying to compensate for deficits. Up to now, the effectiveness of transdiagnostic web-based interventions with either a CAP or a COMP focus has not yet been evaluated.

**Objective:**

This study is the first to examine the effectiveness of two transdiagnostic web-based interventions: (1) the activation of resilience and drawing on existing strengths (CAP: Res-Up!) and (2) the improvement of emotion regulation (COMP: REMOTION), compared with care as usual (CAU) in routine outpatient psychotherapy.

**Methods:**

Adults with at least 1 mental health disorder will be recruited at 4 outpatient centers in Germany. Participants will then be randomized equally into 1 of the 2 intervention groups Res-Up! (CAP) and REMOTION (COMP) or into the control group (CAU). Assessments will be made at baseline (T0), at 6 weeks after treatment start (T1), and at 12 weeks after treatment start (T2). A primary outcome will be symptom severity (Brief Symptom Inventory-18). Secondary outcomes will focus on emotion regulation and resilience.

**Results:**

Participant recruitment and data collection started in April 2020 and were ongoing as of July 2022. We expect participants to benefit more from the interventions than from the CAU control on the dimensions of symptom severity, resilience, and emotion regulation. Furthermore, we expect to find possible differences between CAP and COMP. The results of the study are expected in 2023.

**Conclusions:**

This randomized controlled trial will compare CAU with the transdiagnostic web-based interventions Res-Up! and REMOTION, and will thus inform future studies concerning the effectiveness of transdiagnostic web-based interventions in routine outpatient psychotherapy.

**Trial Registration:**

ClinicalTrials.gov NCT04352010; https://clinicaltrials.gov/ct2/show/NCT04352010

**International Registered Report Identifier (IRRID):**

DERR1-10.2196/41413

## Introduction

### Overview

Approximately 1 in 6 people worldwide have met the criteria for a mental disorder in the last 12 months (17.6%); 29.2% have been identified as having experienced a mental disorder at some time in their life [1]. Mental health problems and disorders have a profound impact on individuals affected by symptoms and society as a whole. Still, in Germany, only 11%-40% of patients receive treatment [2], often due to the long waiting times for psychotherapy (20.1 weeks in 2019) [3]. Therefore, the German mental health care system needs innovative intervention concepts, using approaches beyond standard face-to-face therapy and serving a wide range of mental disorders. Transdiagnostic web-based interventions can provide such an approach [4-6].

### Web-Based Interventions

Recent global developments have fast-forwarded digitalization, and thus the use of web-based tools to support mental health care has rapidly increased all over the world. Research on web-based interventions in psychotherapy supports the effectiveness of different intervention programs for a variety of mental disorders [5,7,8]. With limited access to psychotherapy, web-based interventions are an economical, flexible, and practical alternative or addition to conventional treatment [5]. Particularly when face-to-face psychotherapy is not available because of limited resources (like insufficient availability of therapists), web-based interventions can help to avoid long waiting times for treatment and thus mitigate the chronification of mental health problems [4].

Among web-based interventions, stand-alone web-based programs can be differentiated from blended treatment (BT)—the combination of face-to-face psychotherapy and a web-based intervention [9]. Although BT can take on many different forms [10], a differentiation can be made between integrated blends (eg, conceptual coordination between web-based intervention elements and face-to-face psychotherapy sessions via a specified protocol) and add-on blends (eg, a web-based intervention provided in parallel before or after face-to-face psychotherapy without conceptual coordination or integration between the two). BT, where a web-based intervention was provided as an add-on to face-to-face psychotherapy, has been shown to be more effective than psychotherapy alone [11]. A transdiagnostic approach might be particularly relevant for add-on conceptualizations as coordination with a potential face-to-face therapy focusing on a specific disorder is less relevant.

Although even unguided web-based interventions (without any contact between the patient and the provider of the intervention) seem to be effective, therapist-supported web-based interventions are most likely more effective than self-guided treatments [5,12,13]. Although the exact mechanism behind the benefit of guidance remains unclear, this may be due to higher adherence and lower dropout rates resulting from the positive effect of the accompanying support on motivation and on engagement with the intervention [5,11].

### Capitalization or Compensation: Basic Orientations in Psychotherapy

Overall, a differentiation has been made in psychotherapy research between capitalization (CAP)–oriented versus compensation (COMP)–oriented *interventions*. CAPs aim to draw on the person’s already existing strengths (in a wide sense: action repertoire, resilience strategies, and external and internal resources), and COMPs identify dysfunctional maintenance factors of psychopathology and teach (respectively train) the person to use new strategies or build new behavior against relative deficits [14-18]. In an early study, Wingate et al [18] compared the outcomes of compensation versus capitalization in the treatment of suicidality in young adults, finding results favoring COMPs, whereas other studies find results favoring CAPs [14,17]. According to the current sparse research, there is no agreement on which of these 2 strategies should be pursued in psychotherapy [14,17,19-22].

Certain interventions in psychotherapy are inherently more focused on capitalization (eg, focusing on action repertoire, resilience strategies, and external and internal resources) or on compensation (eg, focusing on the building of new strategies for deficit compensation). Although outcome differences between CAPs and COMPs in face-to-face psychotherapy seem to be small [14], there are no studies comparing the 2 approaches for web-based therapy. Web-based therapy involves less therapeutic guidance and thus relies more strongly on the person’s self-management capacities. Thus, the possible effects of therapists’ support in face-to-face therapy compensating deficits in both CAP and COMP cease in a web setting. Thus, this randomized controlled trial explores, for the first time, the effect of 2 transdiagnostic web-based interventions inherently capitalization or compensation oriented: activation of resilience (CAP) (respectively improvement of emotion regulation [COMP]) compared with a care-as-usual (CAU) group.

As discussed above, capitalization and compensation can be conceptualized as features of interventions. Another possible conceptualization is via the interaction of intervention focus with patient variables. If particularly patients with a high level of adaptive emotion-regulation strategies profit from an intervention focusing on the improvement of emotion-regulation strategies, this would point toward a capitalization change process. If patients with a low level of adaptive emotion-regulation strategies profit particularly from an intervention focusing on the improvement of emotion-regulation strategies, this points to a compensation change process. Thus, in this trial, differential effects of patients’ initial resilience (respectively emotion-regulation competencies) on the outcome of a resilience and emotion-regulation intervention will also be explored.

### Resilience

Resilience as psychological resistance to adversity was originally conceptualized in the context of developmental psychology [23,24]. Today, resilience and resources are often discussed in the field of positive psychology [25], which focuses on factors that make and keep people healthy rather than ill. Although most research on resilience and resources has a compensation focus, aiming to create new resources and building up new strengths [26], the personal model of resilience (PMR) is a CAP intervention that was developed by Padesky and Mooney [27] from a cognitive perspective.

The PMR focuses on individuals’ pre-existing resources and resilient strategies to increase awareness and implementation of these strengths. The basic assumptions of the PMR are as follows: every person is resilient and has resilience strategies that are already part of their action repertoire and have the potential to also be used in challenging situations. The model is based on evidence-based standards for cognitive behavioral therapy [28].

The model was evaluated in a pilot study with students [29], a randomized study with a waiting control group [30], and in a web version with active plus waiting control groups (web-based intervention program vs face-to-face vs ABC [activating events, belief systems, emotional and behavioral consequences] model vs waiting control group) [31]. Medium effect sizes for the face-to-face model and small effect sizes for the web version were observed. Based on the feedback of participants, the web version was redesigned and is being re-evaluated [32]. The redesigned version of the program (Res-Up!: Resilience program of the University of Witten/Herdecke) showed medium effect sizes in preliminary results for resilience (*d*=0.51-0.55) and emotional competence (*d*=0.51), greater effect sizes for self-compassion (*d*=0.70), and small effect sizes for self-esteem (*d*=0.41) [32].

### Emotion Regulation

Emotion regulation refers to the way individuals attempt to influence emotions and includes the upregulation and downregulation of positive and negative emotions in accordance with regulatory goals [33]. Emotion regulation and specifically the fostering of flexible use of different emotion-regulation strategies are important topics in the treatment of mental health disorders and more specifically in psychotherapy research. This is made evident by the large number of publications that focus on the topic of emotion regulation [34-37]. Interestingly, emotion regulation has been discussed as a transdiagnostic factor related to psychopathology and the treatment of mental health disorders [34,38-40]. More recently, several mental health interventions that address or target emotion regulation have been developed as web- or mobile-based programs. For example, Böhme and Berking [41] describe the application of an emotion regulation app based on Affect Regulation Training.

By using the extended process model of emotion regulation as a theoretical framework to structure an emotion-regulation intervention [42], Bielinski et al [43] developed REMOTION. The web-based program aims to reduce the symptom severity of patients with a range of different diagnoses while improving their emotion regulation [43]. REMOTION is currently being examined as a blended intervention in an outpatient psychotherapy setting in a pilot randomized controlled trial [43]. It is also currently being examined as an add-on to acute inpatient psychiatric care in another pilot randomized controlled trial (ClinicalTrials.gov NCT04990674). Results for both trials are expected in 2023.

### Aims

Web-based interventions have been efficient and helpful in considering different mental disorders and in self-selected samples in many controlled studies [7]. Nevertheless, little is known about the efficacy of web-based interventions in routine psychotherapeutic practice and their usefulness as an add-on to conventional psychotherapy (BT).

The *first goal* of this study is to assess the effectiveness of 2 web-based interventions (Res-Up! and REMOTION) in an outpatient psychotherapeutic routine setting in comparison with CAU.

The *second goal* of this study is to explore, in a web-based therapy context, the implementation of interventions with either a CAP or COMP focus by using 2 transdiagnostic web-based intervention programs, centered either on activating resilience (CAP; “Res-Up!”) or on emotion regulation (COMP; “REMOTION”).

Further subanalyses considering differences in patients’ benefits depending on their diagnoses will be conducted. In addition, the differential effects of initial resilience and emotion-regulation competencies on the effectiveness of Res-Up! and REMOTION will be explored. To accomplish this, the initial status of resilience and emotion-regulation competencies will be assessed and related to intervention effects in the intervention groups.

## Methods

### Study Design

The study is a multicenter 3-arm randomized controlled trial in which a CAU approach will be compared with 2 different web-based intervention programs (CAP: Res-Up! and COMP: REMOTION), which will be administered as an add-on to CAU.

Participants in the intervention groups will be given access to Res-Up! and REMOTION immediately, whereas participants in the CAU group will receive access to one of the interventions after 12 weeks (participants in the CAU group can choose which intervention they want to access). Assessments will be made at baseline, 6 weeks (post), and 12 weeks (follow-up) for all participants. Assessments at 6 and 12 weeks will be made irrespective of whether the participant is in face-to-face treatment.

The multicenter trial will take place at the outpatient clinics of the Training Center for Psychological Psychotherapy OWL (Bielefeld, Germany), the Centers for Psychotherapy Dortmund and Muenster (all German Association of Behavioral Therapy—Deutsche Gesellschaft für Verhaltenstherapie [DGVT]), and the Center for Mental Health and Psychotherapy (Department of Psychology and Psychotherapy, University Witten/Herdecke).

Primary and secondary outcomes will be gathered via link to web-based self-report questionnaires based on Qualtrics software [44] at baseline and 6 and 12 weeks after baseline.

**Figure 1:**
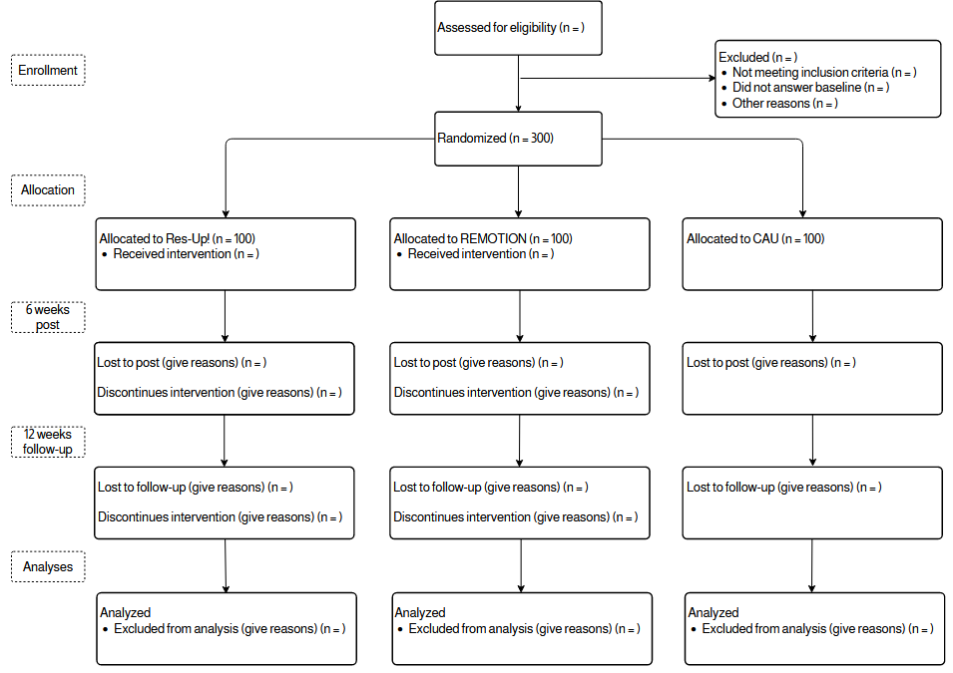
Flow of participants. CAU: care as usual.

All participants will be required to give their informed consent before enrollment in the study. The written consent will be obtained by trained psychotherapists at the outpatient centers. Furthermore, all participants are given the option to contact a study member during the intervention (“guidance on demand”) either via email (REMOTION) or chat (Res-Up!) but are not actively contacted by the team (except for reminders to complete the interventions or questionnaires and a welcome message). Figure 1 displays the flow of patients through the study.

### Sample Size Calculation

Sample size was calculated with the software G*Power [45]. We aim to detect small effect sizes of Cohen *d*=0.20 regarding the Time×Group interaction for the 2 active conditions at an error level of .05. A power analysis revealed that a sample size of 80 participants in each of the active study arms is required to detect a statistically significant difference with a power (1 – β) of .80. The sample size was further estimated based on a dropout rate of approximately 25%. We finally decided to randomize 100 participants to each of the conditions (3 arms, 100 per arm; CAU, Res-Up!, and REMOTION). The second goal of the study is exploratory, and thus no assumptions about differential effects can be made.

### Eligibility Criteria

The inclusion criteria are as follows: (1) current diagnosis of a mental disorder according to *Diagnostic and Statistical Manual of Mental Disorders, Fifth Edition* or *International Classification of Diseases, 10th Revision*; (2) recruitment at one of the participating outpatient clinics listed above; (3) interest to receive psychotherapy at one of the centers but not currently enrolled; (4) at least 18 years of age; and (5) reliable web access. The exclusion criteria are as follows: (1) current severe episode of major depression, (2) current psychotic disorder, (3) acute suicidal tendency, (4) other severe mental disorders (eg, bipolar disorder), and (5) insufficient German language skills. These diagnoses were ruled out because of their severity, as they impair the ability to concentrate on web-based interventions and stay committed for several weeks of programs and measurements. Access to face-to-face psychotherapy after enrollment is not restricted while participants are working with the web-based programs in order to approximate naturalistic conditions.

### Recruitment and Randomization

Recruiting training centers for psychological psychotherapists in Germany are located in Bielefeld, Dortmund, Muenster (training centers of DGVT) and in Witten, Germany (Center of Mental Health and Psychotherapy, Witten/Herdecke University). Trained psychotherapists at all centers will inform interested individuals coming to the institution for a face-to-face consultation session. As waiting lists for psychotherapy are quite long in Germany, the consultation generally does not mark the beginning of psychotherapy but is scheduled to evaluate whether the person has a clinically relevant psychiatric disorder with a structured clinical interview. Interviewers gather written consent to participate in the study, which is forwarded to the study team members. After study eligibility is proven, participants receive an email with a personalized code to the baseline questionnaires. Once participants have filled out the baseline questionnaires, they are randomized via Qualtrics software [44].

### Ethics Approval

The study will be conducted according to local regulations and the Declaration of Helsinki. The study was approved by the ethics committee of the University Witten/Herdecke (221/2019). Written informed consent regarding the gathered data and following analyses as well as scientific publications in an anonymous form will be obtained from all patients by the recruiting therapists in outpatient centers. The trial is registered with ClinicalTrials.gov (NCT04352010). Data will only be analyzed in an anonymous form and will be deidentified by a specific member of the research team.

### Interventions

#### Res-Up!

Res-Up! is a web-based intervention focusing on patients’ strengths and positive experiences developed at the University Witten/Herdecke. The intervention is based on the PMR [27], which is a positive cognitive intervention that uses patients’ strengths to overcome problems. The PMR activates resilient emotions, thoughts, metaphors, images, and behaviors in 4 steps structured into 5 modules. A variety of psychotherapy elements (cognitive behavioral therapy, emotion-focused therapy, positive therapy, etc) are integrated in the program. Participants are asked to work on 1 of 5 consecutive modules per week; completing a module takes about 1-2 hours. Res-Up! is conducted on the web platform Minddistrict [46], which has been approved by the privacy policy office of Witten/Herdecke University before the start of the study. The intervention can be administered as a stand-alone or add-on treatment to psychotherapy. The basic assumption of the PMR is that every individual has a repertoire of resilience strategies that can be used in everyday life or in situations of crisis. Res-Up! aims to identify these strategies and improve, support, and implement them in problematic situations where they were not used before.

In the first module, participants are educated on the concept of resilience. In the second session, individual resilience areas of the participant are elaborated. The goal of the third module is to construct the individual’s current PMR. The PMR is further developed in the fourth module in which resilient strategies from the model are used and adapted for still problematic areas. At the end of this module, a behavioral experiment is planned. The experiment is evaluated in the last module. To further increase participants’ motivation to use their model and resilience strategies, the individual’s experiences with the PMR are summarized.

Training and intervention elements are presented as informational texts as well as interactive modules, individualized questions, videos, and audio files. Throughout the whole intervention, participants can ask for guidance (via chat or email) by a member of the study team who is trained in the program and has at least a master’s degree in psychology. To increase adherence, reminder messages to complete the modules in time are sent via chat. With continuous participation, participants should complete the intervention within 6 weeks.

#### REMOTION

The web-based intervention REMOTION is aimed at reducing symptom severity and improving emotion regulation of individuals with different mental health disorders [43]. The theoretical background and exact structure of REMOTION have been described in detail elsewhere [43]. REMOTION includes an introduction and 5 further modules, and individuals are asked to work on 1 module per week, ideally for around 1-2 hours per week. Intervention elements are presented in different formats including video, text, and audio along with different types of exercises throughout the program. The intervention is accessed via a platform provided by the University of Berne, and the access is password protected. Textbox 1 shows the content of the intervention.

For this study described in this protocol, and to allow comparability with the Res-Up! intervention, REMOTION is administered as a stand-alone or an add-on treatment to psychotherapy. If individuals do not work with the program for an entire week, they are then reminded to work with the program, but no other active guidance components (like weekly emails) are added. Individuals are, however, able to contact the study team via email if they have any questions. Like Res-Up!, completion of the REMOTION program should take around 6 weeks.

**The content of REMOTION shown in detail.**

**Introduction**

- Information about the structure of the intervention and about the theoretical background, and a user guide are provided in this module

**Psychoeducation**

- Information is provided as to what emotions are, what their functions are, and what types of emotional experiences there are
- The concept of emotion regulation is introduced and the relationship between emotion regulation and mental illness is explored

**Identification**

- Emotional awareness, which is identified as key to the perception substep of the identification stage of emotion regulation [42], is explored in this module
- If and when to regulate emotions, along with information on the value of emotion regulation, is introduced in this module

**Selection**

- This module shows patients what types of emotion-regulation strategies are available
- The focus is on the selection of an emotion-regulation strategy [42]
- The strategies situation selection or modification, attentional deployment, cognitive change, and response modulation are introduced in this module [47]
- Furthermore, strategies specific to over- and under-regulated states are also introduced [48-50]

**Implementation**

- This module shows patients how the previously introduced strategies can be implemented, for example, translated to different tactics [42]
- Exercises are introduced for every emotion-regulation strategy, and advice is provided as to how these exercises can be implemented into daily life

**Modification and flexibility**

- The importance of the flexible use of strategies (being able to modify strategies and being able to apply them flexibly) [42,51] is discussed in this module
- Patients are encouraged to flexibly use strategies, to apply them to different contexts, to practice, and to also attempt sequences or blends of strategies that work for them as individuals

Note: The textbox is adapted from Bielinski et al [43] which is published under Creative Commons Attribution 4.0 International License [52].

#### CAU Group

Participants in the CAU group will not get access to the web-based interventions for 12 weeks after baseline. They will answer the questionnaires before randomization as well as 6 (respectively 12) weeks later. They get access to the web-based interventions of their choice after completing the follow-up assessment. During the waiting time, participants are allowed to stay on the wait list or start psychotherapy as they would usually.

### Trial Organization

Participants are being recruited during their registration to regular psychotherapy in the outpatient centers in Germany mentioned above. Recruiting outpatient centers received information about the study from a member of the study team via a short instruction manual, an instruction video, and a personal introduction for psychotherapists in associated centers. Information on the study and the interventions (Res-Up! and REMOTION) is given to interested patients face to face with additional written information by trained psychotherapists. Written consent to participate is solicited by the respective practitioner. Written consent and necessary information (name, birth date, diagnoses, date of recruitment, and email) is given to a member of the study team. Participants then receive an email from the study team with an individualized code to the baseline measure via Qualtrics [44]. Answering individual questionnaires will take approximately 30 minutes at every assessment point. After completing the baseline, participants are randomly assigned by a randomized computer generator (in Qualtrics [44]) to 1 of the 3 treatment arms. As the interventions are planned for 6 weeks, posttreatment measures are provided 6 weeks after baseline. Twelve weeks after baseline, participants are invited to fill out the follow-up measures in order to reach participants with minimal overlap with face-to-face psychotherapy.

Participants in the CAU group are informed that they will have to complete the following two assessments and will get access to the program of their choice after completing the follow-up. In the experimental conditions, participants receive an email with their web access to either (1) Res-Up! (focusing on resilience) or (2) REMOTION (focusing on emotion regulation).

### Measures

#### Overview

Items recording demographic information of patients will be presented at baseline and a reduced battery at 6 and 12 weeks. Patient diagnostic status will be obtained during the initial interview by conducting a Structured Clinical Interview I (German version) for Diagnostic and Statistical Manual of Mental Disorders (respectively a clinical assessment) by an experienced psychotherapist [53,54]. A full description of all outcomes in the study is provided in Table 1. All measures will be provided via a web platform. Data collection will be supported by email reminders.

**Table 1:** Assessments listed by time points.

Variable and outcome			Instrument		Time point				
					Baseline		6 weeks		12 weeks
Demographics					✓		✓		✓
Structured clinical interview or clinical assessment					✓				
**Primary outcome**									
	Symptom severity	BSI-18^a^		✓		✓		✓	
**Secondary outcomes**									
	Resilience	WIRF^b^; CD-RISC-10^c^		✓		✓		✓	
	Emotion regulation	SEK-27^d^; FrAGe^e^		✓		✓		✓	
**Other outcomes**									
	Depressive symptoms	PHQ-9^f^		✓		✓		✓	
	Self-esteem	RSES^g^		✓		✓		✓	
	Self-compassion	SCS-D^h^		✓		✓		✓	
	Working alliance	WAI-I^i^				✓		✓	

^a^BSI-18: Brief Symptom Inventory [55,56].

^b^WIRF: Witten Resource Questionnaire [57].

^c^CD-RISC-10: Connor-Davidson Resilience Scale [58].

^d^SEK-27: Self-assessment of Emotion Regulation Skills [59].

^e^FrAGe: Questionnaire Assessing the Acceptance of Unpleasant and Pleasant Emotions [60].

^f^PHQ-9: Patient Health Questionnaire-9 [61].

^g^RSES: Rosenberg-Self-Esteem Scale [62].

^h^SCS-D: Self-Compassion Scale—German [63].

^i^WAI-I: Working Alliance Inventory I (adapted for web-based interventions) [64].

#### Primary Outcome Measure

The primary outcome measure in this study is general symptom severity measured with the Brief Symptom Inventory—a short form (BSI-18; German version) [55] of the Symptom-Checklist-90-Revised (SCL-90R) [65]. The BSI-18 has 18 items and is a frequently used questionnaire to measure general symptom severity (Cronbach α=.85-.89) with good psychometric properties [56], comparable to those of the SCL-90R [65].

#### Secondary Outcome Measures

*Resilience* will be assessed by two instruments: (1) the Witten Resource Questionnaire [57], a 37-item self-report of personal and external resources with high reliability (Cronbach α=.72-.85), and (2) the Connor-Davidson Resilience Scale-10 [58], an internationally used 10-item self-report of individual resilience with high reliability (Cronbach α=.81-.90).

*Emotion regulation* will be assessed via the following two instruments: (1) Self-assessment of Emotion Regulation Skills-27 (Selbsteinschätzung emotionaler Kompetenzen) [59], a 27-item self-report measure of emotion-regulation skills with high reliability and validity (Cronbach α=.90), and (2) the Questionnaire Assessing the Acceptance of Unpleasant and Pleasant Emotions (Fragebogen zur Akzeptanz von Gefühlen) [60], a 32-item self-report of the acceptance and suppression of pleasant and unpleasant emotions with good reliability and validity.

#### Other Outcome Measures

*Severity of depression* will be assessed with the German version of the Patient Health Questionnaire-9 (PHQ-9) [61]. The PHQ-9 is an internationally used 9-item self-report for screening, diagnosing, monitoring, and measuring the severity of depression with a high retest reliability and validity (Cronbach α>.86).

*Self-esteem* will be assessed with the Rosenberg-Self-Esteem Scale [62], an internationally used 10-item self-report of general self-esteem with high reliability and validity (Cronbach α=.72-.85).

*Self-compassion* will be assessed with the Self-Compassion Scale [63], an internationally used 26-item self-report of self-compassion with high reliability and validity (Cronbach α>.90).

In accordance with meta-analytic results [66], the *therapeutic alliance* between participants and the web-based intervention or study team will be measured with an adapted version of the Working Alliance Inventory for guided Internet Interventions (WAI-I) at the end of the intervention [64]. The WAI-I showed good internal consistency at total and subscale level (Cronbach α between .92 and .94). For the purpose of this study, the term “psychologist” was substituted with the term “study team” in the questionnaire.

Information about whether patients started psychotherapy while working with the web-based interventions will be gathered at 6 weeks and 12 weeks. An overview of the assessments and the measures is provided in Textbox 1. Participants receive up to a maximum of 3 weekly reminders via email if they do not answer the questionnaires in time.

### Planned Analysis

Data will be analyzed using an intention-to-treat (ITT) approach, including all randomized patients in the outcome analyses, and handling missing data accordingly. Participants will be defined as dropouts if no baseline measurement is given. The primary outcome measure, general symptom severity, will initially be analyzed descriptively. The effects of the interventions on the primary and secondary outcome measures will be analyzed with linear mixed-effect models (LMMs). LMMs are recommended for ITT analyses with missing data because of the possibility to accommodate for missing data without having to exclude or impute data and do not depend on limited assumptions about the variance-covariance matrix. Sensitivity analysis will be conducted to analyze the impact of dropouts, psychotherapy, and medication on results. The effect sizes of all within groups for pre- to follow-up changes will be computed as Cohen *d*. In addition, clinical significance of changes will be analyzed (reliable change index) [67].

For categorical data, amount, or percentage will be reported. Significance testing of dichotomous data will be conducted with chi-square tests. Results will be reported in accordance with CONSORT (Consolidated Standards of Reporting Trials) [68] and CONSORT-EHEALTH (Consolidated Standards of Reporting Trials of Electronic and Mobile Health Applications and Online Telehealth) checklists [69].

## Results

Participant recruitment and data collection started in April 2020 and completed in July 2022. Results for the study are expected in 2023. Regarding our research questions, we expect participants of our study to benefit more from the interventions than participants in the CAU control on the dimensions of resilience and emotion regulation, respectively. To accomplish the initial status of resilience and emotion-regulation competencies, they will be assessed and related to intervention effects in the intervention groups. Furthermore, we expect to find possible differences between CAP and COMP when the intervention is delivered via a web platform.

## Discussion

### Principal Findings

This study aims to compare a CAU control with 2 transdiagnostic web-based intervention programs, focusing either on resilience (as an example of capitalization strategies) or on emotion regulation (as an example of compensation strategies).

In accordance with previous research [11], we hypothesize that both interventions will be more efficacious than no additional treatment (CAU). According to Fuhr et al [12], web-based interventions that focus on individual parts of psychotherapy—preferably in a blended approach—might be more effective than all elements of a disorder-specific therapy when implemented in a routine setting. Because of the dearth of studies comparing CAP versus COMP interventions directly, no directed hypothesis can be formulated in this context. While the primary outcome targets symptom severity, secondary outcomes assess resilience and emotion regulation as the specific target areas of the individual interventions.

The study will be conducted in Germany in 4 outpatient centers in a clinical sample of approximately 300 participants, 100 in each of the 3 study arms. Matching the transdiagnostic approach of the interventions, participants in the sample will have a variety of different mental disorders and varying symptom severity.

Results from this study will be valuable for practitioners, patients, and mental health services in all fields, due to the transdiagnostic approach. Furthermore, it will contribute to the knowledge and efficacy of evidence-based web-based intervention programs and possibilities of implementation in outpatient routine care. In particular, it will create new knowledge for web-based interventions focusing on resilience and emotion regulation and thus shine light on possible differences of capitalization- and compensation-oriented interventions in web settings.

### Limitations

There could be several *limitations* that can be considered. First, our results might not be generalizable to the general population of outpatient psychotherapy patients because we will apply exclusion and inclusion criteria and recruit a self-selected sample. Clinical practitioners in outpatient clinics recruit participants and inform in advance to ensure a clinical sample. Second, dropout rates from web assessments due to nonadherence to the interventions are a frequently known problem of web-based interventions [11]. Third, there might be an influence on the results due to Res-Up! and REMOTION being distributed through 2 different platforms, as usability rates might vary. Furthermore, participants have the possibility to contact the study team via email or chat throughout the whole intervention, which should increase adherence to the web-based programs and support motivation [13]. However, it must be taken into consideration that participants were able to reach out to the study team via 2 different methods (chat and email), which might influence adherence. Starting a psychotherapy during participation will be controlled in the study and the analyses. Whether such a combination of web-based interventions and psychotherapy not coordinated with the web-based interventions is regarded as BT or not is a matter of debate in the field [9]. This debate will be addressed in the study publications.

### Conclusions

Therapy Online Plus is a multicenter randomized controlled trial, comparing for the first time in a clinical sample a CAU group with 2 transdiagnostic interventions: Res-Up! and REMOTION. To explore the comparison of the 2 transdiagnostic web-based intervention programs, it will assess the transdiagnostic effectiveness of the above interventions, considering different mental disorders. Furthermore, results will extend existing knowledge about the possibilities of implementing web-based interventions in routine psychotherapy settings.

## Abbreviations

BSI-18: Brief Symptom Inventory-18
BT: blended treatment
CAP: capitalization
CAU: care as usual
COMP: compensation
CONSORT: Consolidated Standards of Reporting Trials
CONSORT-EHEALTH: Consolidated Standards of Reporting Trials of Electronic and Mobile Health Applications and Online Telehealth
DGVT: Deutsche Gesellschaft für Verhaltenstherapie
LMM: linear mixed-effect model
PHQ-9: Patient Health Questionnaire-9
PMR: personal model of resilience
WAI-I: Working Alliance Inventory I (adapted for web-based interventions)

## Data Availability

The data sets generated or analyzed during this study are available from the corresponding author on reasonable request.

## References

[1] Steel Z, Marnane C, Iranpour C, Chey T, Jackson JW, Patel V, Silove D (2014). The global prevalence of common mental disorders: a systematic review and meta-analysis 1980-2013. Int J Epidemiol.

[2] Jacobi F, Höfler M, Strehle J, Mack S, Gerschler A, Scholl L, Busch M, Maske U, Hapke U, Gaebel W, Maier W, Wagner M, Zielasek J, Wittchen H (2014). [Mental disorders in the general population : Study on the health of adults in Germany and the additional module mental health (DEGS1-MH)]. Nervenarzt.

[3] Singer S, Maier L, Paserat A, Lang K, Wirp B, Kobes J, Porsch U, Mittag M, Toenges G, Engesser D (2021). Wartezeiten auf einen Psychotherapieplatz vor und nach der Psychotherapiestrukturreform. Psychotherapeut.

[4] Andersson G, Titov N, Dear BF, Rozental A, Carlbring P (2019). Internet-delivered psychological treatments: from innovation to implementation. World Psychiatry.

[5] Ebert DD, Van Daele T, Nordgreen T, Karekla M, Compare A, Zarbo C, Brugnera A, Øverland S, Trebbi G, Jensen KL, Kaehlke F, Baumeister H (2018). Internet-and mobile-based psychological interventions: applications, efficacy, and potential for improving mental health: a report of the EFPA e-Health taskforce. Eur Psychol.

[6] Titov N, Dear BF, Schwencke G, Andrews G, Johnston L, Craske MG, McEvoy P (2011). Transdiagnostic internet treatment for anxiety and depression: a randomised controlled trial. Behav Res Ther.

[7] Andersson G (2016). Internet-Delivered Psychological Treatments. Annu Rev Clin Psychol.

[8] Andrews G, Cuijpers P, Craske MG, McEvoy P, Titov N (2010). Computer therapy for the anxiety and depressive disorders is effective, acceptable and practical health care: a meta-analysis. PLoS One.

[9] Bielinski LL, Berger T (2020). Internet interventions for mental health: current state of research, lessons learned and future directions. Konsul'tativnaia psikhologiia i psikhoterapiia.

[10] Bielinski LL, Trimpop L, Berger T (2021). [All in the mix? Blended psychotherapy as an example of digitalization in psychotherapy]. Psychotherapeut (Berl).

[11] Berger T, Krieger T, Sude K, Meyer B, Maercker A (2018). Evaluating an e-mental health program ("deprexis") as adjunctive treatment tool in psychotherapy for depression: Results of a pragmatic randomized controlled trial. J Affect Disord.

[12] Fuhr K, Fahse B, Hautzinger M, Gulewitsch MD (2018). [Implementation of an Internet-based self-help for patients waiting for outpatient psychotherapy-first results]. Psychother Psychosom Med Psychol.

[13] Karyotaki E, Efthimiou O, Miguel C, Bermpohl FMG, Furukawa TA, Cuijpers P, Riper H, Patel V, Mira A, Gemmil AW, Yeung AS, Lange A, Williams AD, Mackinnon A, Geraedts A, van Straten A, Meyer B, Björkelund Cecilia, Knaevelsrud C, Beevers CG, Botella C, Strunk DR, Mohr DC, Ebert DD, Kessler D, Richards D, Littlewood E, Forsell E, Feng F, Wang F, Andersson G, Hadjistavropoulos H, Christensen H, Ezawa ID, Choi I, Rosso IM, Klein JP, Shumake J, Garcia-Campayo J, Milgrom J, Smith J, Montero-Marin J, Newby JM, Bretón-López Juana, Schneider J, Vernmark K, Bücker Lara, Sheeber LB, Warmerdam L, Farrer L, Heinrich M, Huibers MJH, Kivi M, Kraepelien M, Forand NR, Pugh N, Lindefors N, Lintvedt O, Zagorscak P, Carlbring P, Phillips R, Johansson R, Kessler RC, Brabyn S, Perini S, Rauch SL, Gilbody S, Moritz S, Berger T, Pop V, Kaldo V, Spek V, Forsell Y, Individual Patient Data Meta-Analyses for Depression (IPDMA-DE) Collaboration (2021). Internet-Based Cognitive Behavioral Therapy for Depression: A Systematic Review and Individual Patient Data Network Meta-analysis. JAMA Psychiatry.

[14] Cheavens JS, Strunk DR, Lazarus SA, Goldstein LA (2012). The compensation and capitalization models: a test of two approaches to individualizing the treatment of depression. Behav Res Ther.

[15] Flückiger C, Caspar F, Holtforth MG, Willutzki U (2009). Working with patients' strengths: a microprocess approach. Psychother Res.

[16] Grawe K, Grawe-Gerber M (1999). Ressourcenaktivierung. Psychotherapeut.

[17] Rude SS, Rehm LP (1991). Response to treatments for depression: the role of initial status on targeted cognitive and behavioral skills. Clin Psychol Rev.

[18] Wingate LR, Van Orden KA, Joiner TE, Williams FM, Rudd MD (2005). Comparison of compensation and capitalization models when treating suicidality in young adults. J Consulting Clin Psychol.

[19] Miller IW, Keitner GI, Ryan CE, Solomon DA, Cardemil EV, Beevers CG (2005). Treatment matching in the posthospital care of depressed patients. Am J Psychiatry.

[20] Sotsky SM, Glass DR, Shea MT, Pilkonis PA, Collins JF, Elkin I, Watkins JT, Imber SD, Leber WR, Moyer J (1991). Patient predictors of response to psychotherapy and pharmacotherapy: findings in the NIMH Treatment of Depression Collaborative Research Program. Am J Psychiatry.

[21] Strauman TJ, Vieth AZ, Merrill KA, Kolden GG, Woods TE, Klein MH, Papadakis AA, Schneider KL, Kwapil L (2006). Self-system therapy as an intervention for self-regulatory dysfunction in depression: A randomized comparison with cognitive therapy. J Consulting Clin Psychol.

[22] Witlox M, Garnefski N, Kraaij V, de Waal MWM, Smit F, Bohlmeijer E, Spinhoven P (2021). Blended Acceptance and Commitment Therapy Versus Face-to-face Cognitive Behavioral Therapy for Older Adults With Anxiety Symptoms in Primary Care: Pragmatic Single-blind Cluster Randomized Trial. J Med Internet Res.

[23] Kobasa SC, Maddi SR (1977). Existential personality theory. Current Personality Theory.

[24] Werner E, Smith R (2001). Journeys from Childhood to Midlife: Risk, Resilience, and Recovery.

[25] Snyder CR, Lopez SJ, Edwards LM, Marques SC (2020). The Oxford Handbook of Positive Psychology.

[26] Bolier L, Haverman M, Westerhof GJ, Riper H, Smit F, Bohlmeijer E (2013). Positive psychology interventions: a meta-analysis of randomized controlled studies. BMC Public Health.

[27] Padesky CA, Mooney KA (2012). Strengths-based cognitive-behavioural therapy: a four-step model to build resilience. Clin Psychol Psychother.

[28] Kuyken W, Padesky CA, Dudley R (2011). Collaborative Case Conceptualization: Working Effectively with Clients in Cognitive Behavioral Therapy.

[29] Victor PP, Teismann T, Willutzki U (2017). A pilot evaluation of a strengths-based CBT intervention module with college students. Behav Cogn Psychother.

[30] Victor P, Dikloo AS, Schwert P, Willutzki U (2018). Resilienz- vs. problemorientierte beratung von studierenden. Z Klin Psychol Psychother.

[31] Victor P, Krug I, Vehoff C, Lyons N, Willutzki U (2018). Strengths-based CBT: internet-based versus face-to-face therapy in a randomized controlled trial. J Depress Anxiety.

[32] Trimpop L, Willutzki U (2021). RES-UP!? Resilient trotz Corona durch online-intervention. Workshopband Psychologie der Arbeitssicherheit und Gesundheit - Ergänzungsband Gewalt in der Arbeit verhüten und die Zukunft gesundheitsförderlich gestalten.

[33] McRae K, Gross JJ (2020). Emotion regulation. Emotion.

[34] Berking M, Wupperman P (2012). Emotion regulation and mental health: recent findings, current challenges, and future directions. Curr Opin Psychiatry.

[35] Gratz KL, Gunderson JG (2006). Preliminary data on an acceptance-based emotion regulation group intervention for deliberate self-harm among women with borderline personality disorder. Behav Ther.

[36] Renna ME, Quintero JM, Fresco DM, Mennin DS (2017). Emotion regulation therapy: a mechanism-targeted treatment for disorders of distress. Front Psychol.

[37] Sheppes G, Suri G, Gross JJ (2015). Emotion regulation and psychopathology. Annu Rev Clin Psychol.

[38] Aldao A, Nolen-Hoeksema S, Schweizer S (2010). Emotion-regulation strategies across psychopathology: A meta-analytic review. Clin Psychol Rev.

[39] Cludius B, Mennin D, Ehring T (2020). Emotion regulation as a transdiagnostic process. Emotion.

[40] Sloan E, Hall K, Moulding R, Bryce S, Mildred H, Staiger PK (2017). Emotion regulation as a transdiagnostic treatment construct across anxiety, depression, substance, eating and borderline personality disorders: A systematic review. Clin Psychol Rev.

[41] Böhme S, Berking M (2021). Entwicklung und Evaluation einer Emotionsregulations-App. Präv Gesundheitsf.

[42] Gross JJ (2015). Emotion regulation: current status and future prospects. Psychological Inquiry.

[43] Bielinski LL, Krieger T, Moggi F, Trimpop L, Willutzki U, Nissen C, Berger T (2020). REMOTION blended transdiagnostic intervention for symptom reduction and improvement of emotion regulation in an outpatient psychotherapeutic setting: protocol for a pilot randomized controlled trial. JMIR Res Protoc.

[44] Suite QR (2022). Qualtrics.

[45] Faul F, Erdfelder E, Buchner A, Lang AG (2009). Statistical power analyses using G*Power 3.1: tests for correlation and regression analyses. Behav Res Methods.

[46] (2022). Minddistrict.

[47] Gross JJ (1998). The emerging field of emotion regulation: an integrative review. Rev General Psychol.

[48] Bohus M, Wolf-Arehult M (2013). Interaktives SkillsTraining für Borderline - Patienten. Das Therapeutenmanual.

[49] Greenberg LS (2015). Emotion-Focused Therapy: Coaching Clients to Work Through Their Feelings, 2nd Ed.

[50] Lynch TR (2018). Radically Open Dialectical Behavior Therapy: Theory and Practice for Treating Disorders of Overcontrol.

[51] Gross JJ, Uusberg H, Uusberg A (2019). Mental illness and well-being: an affect regulation perspective. World Psychiatry.

[52] Attribution 4.0 International (CC BY 4.0). Creative Commons.

[53] Association AP (2013). Diagnostic and Statistical Manual of Mental Disorders (DSM-V).

[54] Wittchen HU, Zaudig M, Skid FT (1999). Strukturiertes klinisches Interview für DSM-IV. Achse I und II. Handanweisung.

[54] Franke G (2000). Brief Symptom Inventory von LR Derogatis(Kurzform der SCL-90-R): Deutsche Version: Testmappe.

[56] Geisheim C, Hahlweg K, Fiegenbaum W, Frank M, Schröder B, von Witzleben I (2002). Das Brief Symptom Inventory (BSI) als Instrument zur Qualitätssicherung in der Psychotherapie. Diagnostica.

[57] Victor PP, Schürmann J, Muermans MM, Teismann T, Willutzki U (2019). Wittener Ressourcenfragebogen (WIRF) – Ein mehrdimensionales Instrument zur subjektiven Ressourceneinschätzung. Zeitschrift für Psychiatrie, Psychologie und Psychotherapie.

[58] Sarubin N, Gutt D, Giegling I, Bühner M, Hilbert S, Krähenmann O, Wolf M, Jobst A, Sabaß L, Rujescu D, Falkai P, Padberg F (2015). Erste Analyse der psychometrischen Eigenschaften und Struktur der deutschsprachigen 10- und 25-Item Version der Connor-Davidson Resilience Scale (CD-RISC). Zeitschrift für Gesundheitspsychologie.

[59] Berking M, Znoj H (2011). SEK-27-fragebogen zur standardisierten selbsteinschätzung emotionaler kompetenzen. LeibnZentrum für Psychologische Information und Dokumentation (ZPID), Electronic Testarchive.

[60] Beblo T, Scheulen C, Fernando SC, Griepenstroh J, Aschenbrenner S, Rodewald K, Driessen M (2011). Psychometric analysis of a new questionnaire assessing the acceptance of unpleasant and pleasant emotions (FrAGe). Z Klin Psychol Psychother.

[61] Kroenke K, Spitzer RL, Williams JB (2001). The PHQ-9: validity of a brief depression severity measure. J Gen Intern Med.

[62] Ferring D, Filipp S (1996). Measurement of self-esteem: findings on reliability, validity, and stability of the Rosenberg Scale. Diagnostica.

[63] NEFF KD (2003). The Development and validation of a scale to measure self-compassion. Self Identity.

[64] Gómez Penedo JM, Berger T, Grosse Holtforth M, Krieger T, Schröder J, Hohagen F, Meyer B, Moritz S, Klein JP (2020). The Working Alliance Inventory for guided Internet interventions (WAI-I). J Clin Psychol.

[65] Derogatis LR, Unger R (2010). Symptom checklist-90-revised. The Corsini Encyclopedia of Psychology.

[66] Flückiger C, Del Re AC, Wampold BE, Horvath AO (2018). The alliance in adult psychotherapy: A meta-analytic synthesis. Psychotherapy (Chic).

[67] Jacobson NS, Truax P (1991). Clinical significance: a statistical approach to defining meaningful change in psychotherapy research. J Consult Clin Psychol.

[68] Schulz KF, Altman DG, Moher D, CONSORT Group (2010). CONSORT 2010 statement: updated guidelines for reporting parallel group randomised trials. BMJ.

[69] Eysenbach G, CONSORT-EHEALTH Group (2011). CONSORT-EHEALTH: improving and standardizing evaluation reports of Web-based and mobile health interventions. J Med Internet Res.

